# Artificial intelligence-assisted visual elicitation in anorexia nervosa: Qualitative case studies

**DOI:** 10.1007/s40211-025-00538-2

**Published:** 2025-07-28

**Authors:** Dimitri Chubinidze, Catherine Perry, Kate Tchanturia

**Affiliations:** 1 Institute of Psychiatry, Psychology and Neuroscience, Department of Psychological Medicine, King’s College London, London, UK; 2 Department of Eating Disorders, South London and Maudsley NHS Foundation Trust, London, UK; 3 Department of Psychology, Illia State University, Tbilisi, Georgia

**Keywords:** Emotion regulation, Visual metaphor, Artificial intelligence-generated images, Guided reflection, Photo elicitation, Eating disorders, Emotionsregulation, Visuelle Metapher, KI(künstliche-Intelligenz)-generierte Bilder, Geführte Reflexion, Fotografische Erkundung, Essstörungen

## Abstract

**Purpose:**

This study explored the feasibility and therapeutic potential of combining artificial intelligence (AI)-assisted visual elicitation with sensory-attuned guided reflection to support emotional expression and engagement in individuals with anorexia nervosa (AN).

**Methods:**

We conducted a two-session, therapist-led intervention with two adults with restrictive AN. In session 1, we guided participants to reflect on emotionally challenging experiences using metaphor and sensory language cues. We translated these narratives into prompts for DALL·E (v3; OpenAI, San Francisco, CA, USA), an AI image-generation tool. In session 2, participants selected from curated images to reflect on and deepen their exploration of emotional experiences. We analysed the data using reflexive thematic analysis and performed a cross-case synthesis.

**Results:**

Visual metaphors helped to externalise and communicate emotions, evoke embodied responses and refine affective descriptions. The co-creative process fostered therapeutic engagement and a greater sense of agency in both participants.

**Conclusion:**

AI-assisted visual elicitation, integrated into a structured therapeutic process, may offer an additional benefit to talking therapy for individuals with AN. By enabling visual expression, this approach could support emotional insight and communication in populations with emotion labelling and regulation differences.

## Abstract (de)

**Zielsetzung:**

Diese Studie untersuchte die Machbarkeit und das therapeutische Potenzial der Kombination von KI(künstliche Intelligenz)-gestützter visueller Elizitation mit sensorisch abgestimmter geführter Reflexion zur Unterstützung von emotionalem Ausdruck und Engagement bei Personen mit Anorexia nervosa (AN).

**Methoden:**

Wir führten eine therapeutengeleitete Intervention in 2 Sitzungen mit 2 Erwachsenen mit restriktiver AN durch. In der ersten Sitzung bewegten wir die Probanden, über emotional herausfordernde Erlebnisse zu reflektieren, indem wir Metaphern und sensorische Stichworte verwendeten. Wir übersetzten diese Erzählungen in Prompts für DALL‑E (v3; OpenAI, San Francisco/CA, USA), ein KI-Tool zur Bilderzeugung. In der zweiten Sitzung wählten die Probanden unter kuratierten Bildern aus, um über ihre emotionalen Erfahrungen zu reflektieren und diese zu vertiefen. Wir analysierten die Daten mithilfe einer reflexiven thematischen Analyse und führten eine fallübergreifende Synthese durch.

**Ergebnisse:**

Visuelle Metaphern halfen dabei, Emotionen zu externalisieren und zu kommunizieren, verkörperte Reaktionen zu evozieren und affektive Beschreibungen zu präzisieren. Der kokreative Prozess förderte das therapeutische Engagement und stärkte das Gefühl von Handlungsfähigkeit bei beiden Beteiligten.

**Schlussfolgerung:**

Integriert in einen strukturierten therapeutischen Prozess kann die KI-gestützte visuelle Elizitation einen zusätzlichen Effekt zur Gesprächstherapie für Menschen mit AN bieten. Indem er den visuellen Ausdruck ermöglicht, könnte dieser Ansatz die emotionale Einsicht und Kommunikation in Gruppen mit unterschiedlicher Emotionsmarkierung und Emotionsregulation unterstützen.

## Introduction

Anorexia nervosa (AN) is a severe eating disorder (ED) characterized by intense fear of weight gain and persistent restrictive eating, but its psychopathology extends beyond food and body image disturbances. In particular, patients often exhibit profound difficulties in emotional processing—they struggle to recognize, label, interpret and express their own feelings and others’ emotions [1–3]. Many individuals with AN have high levels of alexithymia, indicating difficulties to identify or describe emotions [4]. They often suppress their emotions, using fewer descriptive words and displaying more limited facial expressions compared to individuals without ED [5]. At the same time, sensory processing difficulties are increasingly recognized in AN: patients report heightened sensitivity to various sensory cues [6]. This sensory sensitivity can amplify emotional distress in everyday situations and contribute to the well-known psychosomatic complexity of AN [3, 5, 7–11]. Taken together, these findings point to the intertwined emotional and sensory challenges in AN, highlighting a potential need for adjunct approaches that more directly support emotional awareness, communication and engagement—particularly for individuals who may not fully benefit from talking therapies.

Recently, there has been a growing emphasis on addressing the emotional difficulties associated with AN. One example is Cognitive Remediation and Emotion Skills Training (CREST), developed as an adjunct therapy to improve patients’ cognitive flexibility as well as emotion recognition and regulation [12–14]. However, interventions like CREST have limitations. For instance, commonly used emotion assessment tools—such as interviews and questionnaires—may not capture the nuanced, embodied emotional experiences reported by AN patients. Enabling patients to express emotions and sensations through more accessible and diverse modalities may improve communication, mutual understanding and therapeutic engagement.

### Visual metaphors for emotional reflection

Metaphors offers a powerful bridge between internal experience and shared understanding [15]. Visual metaphors in particular can help externalise complex emotional states and promote communication between patient and therapist, even when verbal expression is limited [16, 17]. Structured and collaborative metaphor-based approaches can scaffold this process and make it more accessible.

Arts-based interventions such as Focal Integrative Arts Psychotherapy (FIAP) integrate narrative, sensory engagement and visual tools to support emotional insight. Preliminary evaluations suggest that such approaches are especially valuable for individuals with multifaceted, comorbid presentations [18]. Sensory ethnographic research similarly supports the integration of multimodal, embodied techniques in understanding the lived experience of AN [19]. Recent participatory research using photovoice has also demonstrated the value of inclusive, image-based methods for identifying research priorities with autistic people with experience of ED, further supporting the relevance of visual and arts-based approaches in this population [20].

One technique consistent with these multimodal approaches is visual elicitation, which uses visual materials to facilitate emotional reflection. Visual elicitation involves using images or multimedia as prompts to evoke emotions and memories during therapy; this technique can encourage patients to express themselves through means that offer an alternative to verbal dialogue. By reacting to or even creating visual content, AN patients might externalize feelings that are otherwise difficult to verbalize, leading to more meaningful dialogues about their emotional world. Research demonstrates that images can elicit profound emotional responses, highlight significant themes and enhance communication, particularly in sensitive or emotionally charged contexts [21–25]. Building on the use of visual metaphors in therapy, AI-assisted image generation provides a novel tool for co-creating emotionally resonant imagery with individuals who experience difficulties in verbal expression.

### AI-assisted visual elicitation in therapeutic contexts

Artificial intelligence (AI) is introducing new possibilities for therapeutic engagement, particularly in supporting emotional expression through nonverbal means. One emerging application is visual elicitation using AI-generated imagery based on individuals’ descriptions. These visuals can help externalise emotional states and transform subjective experiences into tangible, shared representations. Comparable developments in AI-enhanced installation art show that multisensory, emotionally responsive environments can foster reflection and deepen emotional engagement [26].

AI-assisted art therapy is beginning to adopt such technologies, including generative algorithms and immersive tools. These approaches show potential in supporting emotional processing for individuals who find verbal expression challenging. Digital creative environments have been found to promote engagement, autonomy and communication [27]. A recent review also highlights AI’s potential to increase access to art therapy, especially for those with limited motor skills or difficulties attending in-person sessions [28]. Evidence from group-based art therapy further shows that digital media can enhance self-confidence, facilitate emotional expression and support sensory regulation in populations with communication difficulties [29].

While AI offers new possibilities for emotional expression in therapy, its use raises ethical and relational considerations [30]. Therapist-guided approaches—such as visual elicitation based on co-created prompts—can help ensure emotional safety, relevance, personalisation and collaborative meaning-making, particularly for individuals with communication difficulties.

### Study rationale

To address the need for accessible, emotionally expressive interventions for AN, this study builds on prior sensory ethnographic research [19] to examine the feasibility and relevance of an AI-assisted visual elicitation technique in the context of AN. The aim is to explore how co-created, image-based reflection may support emotional awareness and therapeutic engagement, particularly for individuals who face challenges with emotional expression.

## Methods

### Participants

We recruited two adult patients, each with a clinical diagnosis of AN (restrictive subtype), from a specialist ED outpatient service. To protect anonymity, we refer to the participants as Patient 1 (P1) and Patient 2 (P2). The clinical team made the diagnoses at the time of admission. We collected the data as part of a broader sensory ethnographic project, approved by the local NHS Research Ethics Committee (ref.: 23/LO/0698). Both individuals had reported communication needs, as outlined in their communication passports, and indicated a preference for expressing emotions through visually descriptive language and other creative forms of expression, such as drawing pictures to illustrate feelings when verbal communication proved difficult.

Data were collected during the middle phase of each participant’s ongoing talking therapy, allowing for integration of the AI-assisted intervention within an already established therapeutic relationship. At the time of the study, P1 was receiving sertraline as prescribed medication, while P2 was not taking any psychiatric medication. Both participants were underweight, reflecting the clinical presentation and diagnostic criteria of AN. However, they did not provide consent to share body weight or body mass index (BMI) values; therefore, no numerical indicators of weight are included in this paper.

Each participant worked with a female therapist who had lived experience of ED and identified as neurodivergent. P2 received a personalised version of the Maudsley Model of Anorexia Nervosa Treatment for Adults (MANTRA), tailored to her sensory and cognitive profile. P1 received an integrative therapeutic approach combining elements of MANTRA, cognitive behavioural therapy (CBT) and sensory-focused techniques, all informed by the PEACE Pathway framework. These adaptations reflected the clinical aim of providing neurodivergence-affirming care within an ED service.

Informed consent was obtained from both participants, covering the use of anonymised qualitative data for scientific publication and dissemination.

### Study design and procedure

This qualitative pilot study explored the use of AI-assisted visual elicitation, integrated with sensory-attuned guided reflection, to support emotional articulation and therapeutic engagement in individuals with AN. Building on prior sensory ethnographic research [19] the study applied a structured two-session protocol. Each session lasted approximately 50–60 min and was audio recorded, transcribed verbatim and anonymised prior to analysis.

#### Session 1. Sensory-attuned guided reflection

Participants took part in a semi-structured therapeutic session centred on emotionally challenging experiences. Using open-ended prompts, they were invited to describe these experiences through sensory language cues (e.g. colour, texture, shape, temperature, spatiality). This process aimed to externalise emotional states through embodied and symbolic expression.

#### AI-assisted visual elicitation

Between Session 1 and 2 participant narratives were translated into text prompts for DALL·E (version 3; OpenAI, San Francisco, CA, USA; 2023), an AI image-generation tool. DALL·E is a deep learning model developed by OpenAI that generates images from natural language descriptions. It uses a version of the Generative Pre-trained Transformer (GPT) architecture, trained on a large dataset of image–text pairs, enabling it to produce novel, high-resolution images that visually represent user-provided prompts. For each emotional theme, 3–8 images were generated. The images were curated by the research team to closely reflect participants’ metaphorical descriptions and maximise emotional resonance.

#### Session 2. Image-based exploration and reflection

In a follow-up session, the AI-generated images were used as visual prompts to facilitate emotional reflection. Participants explored which visual representations resonated with or diverged from their lived experience. Optional drawing and annotation materials were provided to deepen engagement, shared understanding and support expressive meaning-making.

### Reflexivity

Reflexivity was integrated throughout the study as a collaborative process between the therapist and researcher. Following each session, the therapist completed structured reflexive notes documenting participant engagement, shifts in emotional articulation, therapeutic adaptations (e.g. pacing, use of visual tools) and personal clinical reflections. These notes informed the ongoing interpretation of the process and helped contextualise the therapeutic dynamics, though they were not formally analysed as primary data.

The generation and curation of AI-generated images was conducted collaboratively. Based on participants’ metaphorical and sensory descriptions, the authors produced a range of images using DALL·E. The therapist, who had direct, significant clinical knowledge of each participant, reviewed and selected images for use in the second session. This step was essential to ensure emotional safety, ethical appropriateness and alignment with the therapeutic context. Images considered potentially misaligned, or ambiguous were excluded prior to participant exposure.

### Analysis

We conducted reflexive thematic analysis [31] to explore how participants engaged with the guided reflection and AI-assisted visual elicitation process, and to identify difficulties in emotional insight gained across the two therapy sessions. Analysis was conducted sequentially to allow comparison between Session 1 and Session 2 for each participant.

The analysis was conducted using Braun and Clarke’s reflexive thematic analysis approach [31], focusing on sensory metaphors and emotional content across therapy sessions.

Following individual case analysis, a cross-case synthesis was conducted to explore patterns of convergence and divergence in therapeutic engagement and emotional articulation.

In the following section, findings for P1 and P2 are presented separately, followed by a case synthesis.

## Results

### Patient 1

#### Background

P1 is an 19-year-old heterosexual woman of mixed ethnic background, with a six-year history of restrictive AN and comorbid diagnoses of autism and anxiety, made by specialist services prior to her admission to the ED service. She lives with her mother. Clinically, she presented with longstanding difficulties in emotional awareness and expression, describing a sense of emotional blurring since adolescence and a pervasive disconnection from her internal states. She reported relational strain and expressed somatic distress and high anxiety following food intake.

Although no formal diagnosis of depression was recorded, the patient expressed low mood, withdrawal and hopelessness. These challenges in identifying, verbalising and understanding emotions informed the therapeutic focus of the sessions, which aimed to aid emotional articulation.

#### Session 1

P1 chose to explore an emotional state she referred to as “feeling dead”, which she described as central to her everyday experience. As she reflected on the sensory qualities of this state and considered how it might be represented visually—as a sensory object—she and the therapist collaboratively differentiated three emotional states. Each was articulated through a metaphorical expression: “trapped in a box”, “feeling dead” and “an endless tunnel”.

##### Trapped in a box

reflected a sense of emotional containment and disconnection. P1 described her body as a hard, impenetrable shell: “Like my body is the box, and I’m inside it … it just feels quite hard and thick. If you saw it, you’d think, ‘Oh, it’s strong.’” She also mentioned the somatic dimension of this feeling and described it as a physically tiring weight.

##### Feeling dead

was described through greyness and numbness, conveying the dissociative nature of the experience: “It feels like I’m trapped in myself, looking out … like I’m seeing the world from an empty body.” She likened herself to an observer of her own life, describing her actions as “automotions”: “It doesn’t feel like I’m the one doing it.”

##### The tunnel

metaphor captured a sense of disorientation and internal division: “It’s like a tunnel that never ends … one version of me is running, the other is just standing still.” This duality reflected the contrast between functional daily behaviours and emotional paralysis. The tunnel was described as featureless and unchanging: “There’s no end.” These metaphors combined spatial, visual and tactile elements to form an embodied emotional vocabulary. Each of the three themes was translated into a text prompt, and several corresponding images were generated using DALL·E.

#### Session 2

**Fig. 1:**
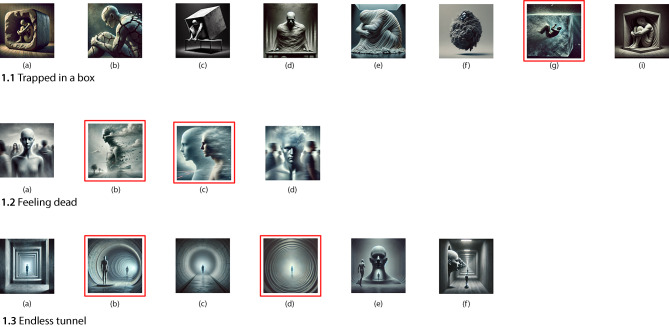
AI-generated images for “Trapped in a box,” “Feeling dead” and “Endless tunnel”. This figure displays AI-generated images created in response to P1’s sensory metaphors of emotional states during Session 1 (Red square outlines indicate the images P1 selected as best representing her experience)

In the second session, P1 engaged with a set of curated images that visually represented the emotional states explored in Session 1. These visual prompts supported further reflection and opened up more specific emotional layers. Below, we outline the additional insights that emerged through this visually supported process. An overview of the metaphors and their development across sessions is provided in Table 1, which illustrates how symbolic language evolved through visual engagement. Figure 1 presents AI-generated images representing key emotional themes, with participant-selected visuals marked by red square boxes.

**Table 1:** Visual metaphor co-development for each participant across session 1 and 2

Participants	Session 1 Metaphors	Session 2 Refinements
Patient 1	Trapped in a box	Dynamic sinking
	Feeling dead	Emotional magnification
	Endless tunnel	Tunnel as dissociative backdrop
Patient 2	Rubbish tip landslide	Toxic engulfment
	Force-fed funnel	Loss of agency
	Flowing blue substance	Shaping texture
	Heavy clouds	Density and distinction

##### From static to dynamic entrapment

The image that resonated most depicted a figure sinking within a translucent, water-filled cube (Fig 1.1g). P1 recognised this as closely aligned with her internal experience: “That’s how ‘trapped’ looks in my mind … like me just sinking.” While her earlier metaphor described a static, enclosed state, this image introduced a sense of downward motion and emotional overwhelm: “It’s like I’m sinking down and down.”

##### Magnification of emotion

P1 also responded to the image’s use of contrast and brightness, noting: “That part is the main focus—like how I’m feeling is magnified.” (Fig. 1.2c). This prompted reflection on how emotional intensity can become overwhelming in daily life, drawing attention to moments when affective experiences feel enlarged or difficult to contain.

##### Differentiating chronic and episodic states

**Fig. 2:**
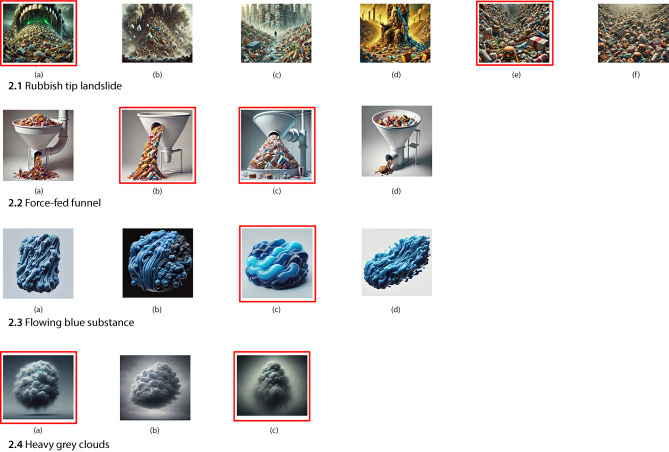
AI-generated images for “Rubbish tip landslide”, “Force-fed funnel”, “Flowing blue substance” and “Heavy clouds”. This figure displays AI-generated images created in response to P2’s sensory metaphors of emotional states during Session 1 (Red square outlines indicate the images P2 selected as best representing her experience)

Images associated with “feeling dead” helped P1 distinguish between more persistent and situational emotional experiences. She selected fog-like depictions of dissolving figures (Fig. 1.2b and 2c) and commented: “It’s less dominant than feeling completely emotionally dead. That’s more constant.” This marked a subtle but meaningful shift in how she differentiated layers of emotional detachment.

##### The tunnel as emotional backdrop

P1 selected images of long, monochromatic corridors (Fig. 1.3b and 3d), which evoked the sense of an ongoing, dissociative background to her life: “I’m just this figure standing there … all that’s behind me is the tunnel.” These visuals helped her express how emotional detachment shapes memory and social engagement: “It’s like the tunnel is there even when I don’t notice it.”

P1 also reflected on how the use of visual and collaborative tools contributed to a sense of therapeutic safety and connection: “You’ve always adapted things—like suggesting I draw instead of writing. That’s how I express myself best.”

#### Case summary

In P1’s case, the use of visual metaphors helped differentiate persistent from situational emotional states. AI-generated imagery supported a reframing of internal experiences, particularly shifting metaphors from static to dynamic forms. The collaborative process facilitated articulation of dissociation and emotional overwhelm, while the collaborative format enhanced therapeutic communication and emotional engagement.

### Patient 2

#### Background

P2 is a 32-year-old heterosexual woman of White British background, with a ten-year history of restrictive AN. Although no formal comorbidities were recorded at the time of admission assessment, she scored above the threshold on the AQ-10 autism screening tool [32], and an assessment for possible attention-deficit/hyperactivity disorder (ADHD) is ongoing. Clinically, she presented with emotional exhaustion, chronic fatigue and difficulties verbalising emotional states. She described herself as a perfectionist and reported feeling overwhelmed by internal and external expectations, often expressing a sense of not doing things “right”. While depression was not formally diagnosed, her presentation—marked by low energy, fatigue and a sense of failure.

She experiences gastroparesis-related symptoms, including nausea and involuntary vomiting, which contribute to her restrictive eating. A history of COVID-19 infection further disrupted her food intake. Raised by a mother who also lives with chronic fatigue and restrictive eating patterns, P2 described a home environment where mental health concerns were often minimised and framed in physical terms.

These emotional and relational challenges shaped the therapeutic focus of the sessions, which aimed to support emotional communication.

#### Session 1

P2 chose to explore the affective state of feeling constantly overwhelmed. Through collaborative reflection with the therapist, this experience was articulated through four metaphorical representations: “rubbish tip landslide”, “force-fed tunnel”, “flowing blue substance” and “heavy clouds”. While these metaphors described related aspects of the same underlying state, the reflective process helped differentiate specific emotional qualities within it, giving each metaphor distinct features while retaining areas of overlap.

##### Rubbish tip landslide

P2’s primary metaphor described being buried under a vast rubbish tip filled with discarded food. She spoke of a sensation of suffocation and drowning: “I feel like I’m suffocating and drowning in a sea of food.” This visual scene appeared particularly in relation to eating during structured mealtimes and was associated with a physical sensation of chest pressure that made it hard to breathe. The rubbish contained unfamiliar foods—such as burgers and snacks—that made the scene feel invasive and beyond her control.

She explained that the experience typically began gradually, with small amounts of food, and then escalated rapidly: “It starts slowly—one or two pieces at first—but then truckloads all at once.” The visual metaphor of a landslide paralleled her emotional buildup, where minor triggers could result in a sudden loss of control. Once overwhelmed, she described the state as “fixed and inescapable”.

##### Force-fed funnel

Another metaphor illustrated the experience of being forced to eat under pressure from others. P2 imagined this as being pushed through a narrow plastic funnel: “It’s like being pushed through a funnel, being choked and forced down it.” She compared it to the practice of force-feeding animals in foie gras production: “Like a goose being force-fed … they fatten them up to make them taste better. That’s what it feels like.” This metaphor captured her feelings of bodily violation, external pressure and helplessness.

##### Flowing blue substance

The metaphor of a flowing blue substance emerged through guided reflection, as P2 attempted to describe the overwhelming and shifting nature of her frustration and self-criticism. She explained: “It’s blue … like water, but not water. It’s rushing and cascading.”

##### Heavy clouds

Finally, P2 described emotions such as sadness and anger as heavy, grey clouds. “They’re like winter clouds—heavy and bleak, but if you get close, you can see through them.” She began to differentiate between them—sadness as more transparent and passive, and anger as heavier and harder to contain.

#### Session 2

In the following therapy session, P2 engaged with curated images to further explore the emotional states identified in the previous session. The visual process elicited strong embodied responses, including somatic sensations such as nausea and tightness. These responses created an opportunity to clarify, revise or deepen her awareness of internal states. Refer to Table 1 for an overview of metaphor development across sessions. Figure 2 presents AI-generated images representing key emotional themes.

##### Rubbish tip landslide: from overwhelm to toxicity

When revisiting her metaphor of the rubbish tip, P2 selected images that depicted an oversized open mouth looming over mountains of rotting food waste, tinted with a fluorescent green glow (Fig. 2.1a; 1e). She responded: “It just feels quite toxic … Like that horrible green.” The visual addition of colour and exaggerated proportions deepened the metaphor, shifting its meaning from mere overwhelm to contamination and toxicity. She further described the scene as emitting a gas-like threat: “It feels like a sort of toxic gas that’s just going to build up inside me.”

Another selected image showed a narrow, landfill-like corridor filled with brightly coloured processed foods. The depth and repetition in the image evoked a sense of being engulfed and unable to escape: “It goes on and on. No end in sight.” Through visual reflection, the metaphor evolved to include sensations of nausea, pollution and claustrophobic entrapment—highlighting a visceral, multisensory quality that extended beyond verbal articulation.

##### Force-fed funnel: loss of control

Images related to the “force-fed funnel” metaphor (Fig. 2.2) further clarified P2’s feelings of violation and restricted agency. She rejected some options (Fig. 2.2a and 2d), explaining: “There’s too much freedom … I don’t like that they could get out of that.” Instead, she selected images (Fig. 2.2b and 2c) showing rigid white funnels force-feeding processed foods into narrow, compressed spaces.

Pointing to image 2c, she remarked: “I would be like that little burger in the middle. And all the food would just be piling into me.” This revised metaphor placed her inside the mechanism itself, highlighting a heightened sense of passivity and entrapment. The visual representation reinforced the unidirectional, coercive nature of the experience—emphasising her lack of choice and control over her body. While the “rubbish tip” illustrated emotional overflow from within, the “funnel” metaphor captured external pressure and systemic force. Together, these images revealed two distinct pathways to the same emotional endpoint: the loss of control—whether through excess or imposition.

##### Flowing blue substance: shaping frustration

Image-based reflection helped P2 refine this metaphor further by engaging more directly with sensory and spatial features. She selected image c (Fig. 2.3c), explaining: “That’s the bit that’s not really landing—the texture … What I see is a bit more fluid—it doesn’t really have a shape.” Although the image was not a perfect match, it enabled her to revisit and rearticulate the emotional texture of frustration. She reaffirmed the shifting sensation: “At first, it’s thick and goopy … then it starts thinning out.” She also noted the absence of rigid contours, indicating that her frustration lacked clear boundaries.

##### Heavy clouds: density and distinction

The final set of images visualised P2’s heavy cloud metaphor (Fig. 2.4a; 4c). She selected images (a) and (c), noting how they captured the emotional presence of sadness: “It would be more of a definite sphere … I can actually see it—like, there’s a sadness there.” She described these cloud-like emotions as static and unchanging: “No, those stay the same—they don’t shift until they’re gone.”

The visual reflection helped P2 clarify the metaphor’s internal structure, differentiating emotional states by density, movement and visibility. She identified sadness as a dense but visible presence, and anger as a force of internal pressure.

#### Case summary

In P2’s case, visual imagery intensified her emotional awareness and evoked embodied responses, including somatic sensations such as nausea and tightness. The process of refining her metaphors supported therapeutic engagement and contributed to a growing sense of agency. As she differentiated between emotional textures, her ability to describe and reflect on her internal states improved. The contrast between fixed emotional states, such as sadness and anger, and more fluid ones, like frustration, further supported her capacity to distinguish and articulate nuanced affective experiences.

#### Cross-case synthesis: emotional articulation through visual elicitation

We synthesised findings across the two cases and identified four shared therapeutic processes. These mechanisms—externalising emotions, embodied emotional resonance, collaborative meaning-making and differentiating emotional experience—capture how sensory-attuned reflection, combined with AI-assisted visual elicitation, supported emotional processing, therapeutic engagement and connection. Below, we briefly introduce each theme and illustrate them with case examples, summarised in Table 2.

**Table 2:** Cross-case themes and metaphor development through visual elicitation

Theme	Patient 1	Patient 2
Externalising emotions	Tunnel imagery gave concrete form to dissociative detachment	Rubbish tip imagery represented emotional overwhelm and loss of control
Embodied emotional resonance	Described emotional states as physically tiring and heavy	Reported nausea, tightness and sensory overload during image exploration
Collaborative meaning-making	Recognised therapist’s adaptation of visual tools to match communication style	Revised funnel metaphor to reflect internalised passivity and lack of agency
Differentiating emotional experience	Distinguished fluctuating vs. persistent emotional states (e.g. trapped vs. feeling dead)	Differentiated texture and movement across frustration, sadness and anger

This table highlights how each participant expressed and revised emotional metaphors across sessions, illustrating shared therapeutic mechanisms despite individualised experiences

#### Externalising emotions

In this context, externalising refers to the process of giving visual or sensory form to internal emotional experiences that are difficult to articulate verbally. AI-generated images acted as mediators between felt experiences and symbolic expression. For P1, the tunnel metaphor validated her dissociation; for P2, vivid imagery triggered embodied reactions, reinforcing emotional presence. These visual tools served as bridges between felt but unspoken experiences and symbolic articulation.

#### Differentiating emotional experience

This refers to participants’ increasing capacity to distinguish between emotional states in terms of quality, intensity and temporal patterning. Through visual engagement, P1 was able to differentiate between persistent and episodic states (e.g. deadness vs. entrapment), while P2 distinguished emotional textures—describing frustration as fluid, sadness as dense and anger as eruptive. These refinements contributed to enhanced emotional clarity and regulation potential.

#### Embodied emotional resonance

Embodied emotional resonance captures how emotional states are not only cognitively processed but also physically felt. The AI-generated visuals elicited bodily sensations that reflected participants’ affective experiences. P1 described emotional exhaustion as weight and fatigue, while P2 experienced nausea and tightness during image exploration. This deepened their emotional connection and reinforced therapeutic insight.

#### Collaborative meaning-making

This theme reflects the co-construction of meaning between therapist and participant through shared metaphor development and visual exploration. Both participants contributed actively to shaping their emotional representations. P1 responded to therapist flexibility in using drawing and visual tools; P2 revised her metaphors based on the imagery, reframing her funnel metaphor to better reflect internalised passivity and lack of agency.

## Discussion

This study explored how AI-assisted visual elicitation, integrated with guided sensory reflection, supported emotional awareness and therapeutic engagement in individuals with AN. The intervention was tested in response to known challenges for many individuals with AN, including difficulties in recognising, differentiating and communicating emotional states—often compounded by alexithymia, sensory challenges [4, 7, 10].

### Enhancing emotional awareness through guided AI-enhanced guided reflection

We invited participants to reflect on emotionally difficult states through sensory-based prompts during a structured therapeutic session. This process supported the generation of metaphorical language—descriptions grounded in texture, space, temperature and movement—that represented affective experiences. We then translated these participant-generated metaphors into image prompts, which informed the AI-assisted image generation process. This technique builds on previous work [19], where similar techniques were used to explore the lived sensory experiences of individuals undergoing treatment for AN.

We curated all images during the generation process, drawing on clinical insight and participant narratives, rather than relying solely on automated or interpretive methods. In the second session, participants engaged with these visuals to explore, select and reframe representations that resonated with their emotional experiences. This guided, iterative process—combining therapist-facilitated reflection with AI-assisted photo elicitation—supported the articulation of more differentiated emotional states, such as shifts in intensity, temporality and embodied expression. These developments reflect increased emotional granularity and communication, both of which are associated with improved emotion regulation and therapeutic outcomes [3, 14].

### Engagement and agency through digital co-production

The use of AI-generated images was reflective and participatory. Patients engaged actively with the visual material, commenting on its accuracy, rejecting mismatched representations and collaborating with the therapist to revise emotional metaphors. This co-creative dimension enhanced therapeutic engagement, as participants took ownership of the representational process and used it to communicate emotions that were previously difficult to verbalise. As found in other digital and arts-based interventions, such interaction can foster autonomy, increase relevance and reduce the emotional demand of verbal expression alone [18, 29].

Therapist mediation was essential throughout this process. While AI tools provided the generative material, it was the therapist’s attuned responses—adapting to communication styles, supporting emotional pacing and guiding reflection—that sustained the therapeutic value of the intervention. This resonates with emerging findings on AI in therapy, where relational framing and ethical use are critical to maintaining emotional safety and therapeutic clarity [30].

### Clinical implications and ethical considerations

The process demonstrated here offers a potential adjunct to talking therapies for AN, particularly for patients who find direct verbal reflection challenging. AI-assisted visual elicitation may expand the therapeutic tools by supporting patients’ creative capacities, contributing to more inclusive, personalised and engaging modalities for enhancing emotional awareness and communication.

However, the integration of AI tools in therapy requires careful ethical consideration. As previous research highlights, risks include emotional misalignment, relational confusion and over-reliance on digital outputs [27, 28]. In this study, the therapist and research team mitigated these risks by embedding AI use within a relational, co-productive and clinically informed process. The imagery was considered as a prompt for dialogue and reflection.

### Limitations

With only two participants, the findings are not generalisable to broader or more diverse populations. The AI tool used was not developed for therapeutic purposes, and individual responses to the imagery may vary. These are just a few of the factors that limit the study’s broader applicability.

## Conclusion

Artificial intelligence (AI)-assisted visual elicitation, when embedded in a structured therapeutic framework, can support emotional awareness and engagement in individuals with anorexia nervosa (AN). Through the integration of sensory-attuned guided reflection with AI-assisted visualisation, participants were able to externalise, differentiate and communicate complex emotional experiences. These findings highlight the potential of multimodal, participatory approaches for individuals with communication and emotion regulation difficulties and encourage further exploration of ethically grounded uses of AI in mental health care.
